# Supplementary material for: The Polyubiquitin Gene MrUBI4 Is Required for Conidiation, Conidial Germination, and Stress Tolerance in the Filamentous Fungus Metarhizium robertsii
Source: Genes (Basel). 2019 May 29;10(6):412. doi: 10.3390/genes10060412 (PMC6627135; doi:10.3390/genes10060412)
Supplement: Supplementary file 1 [file genes-10-00412-s001.pdf]

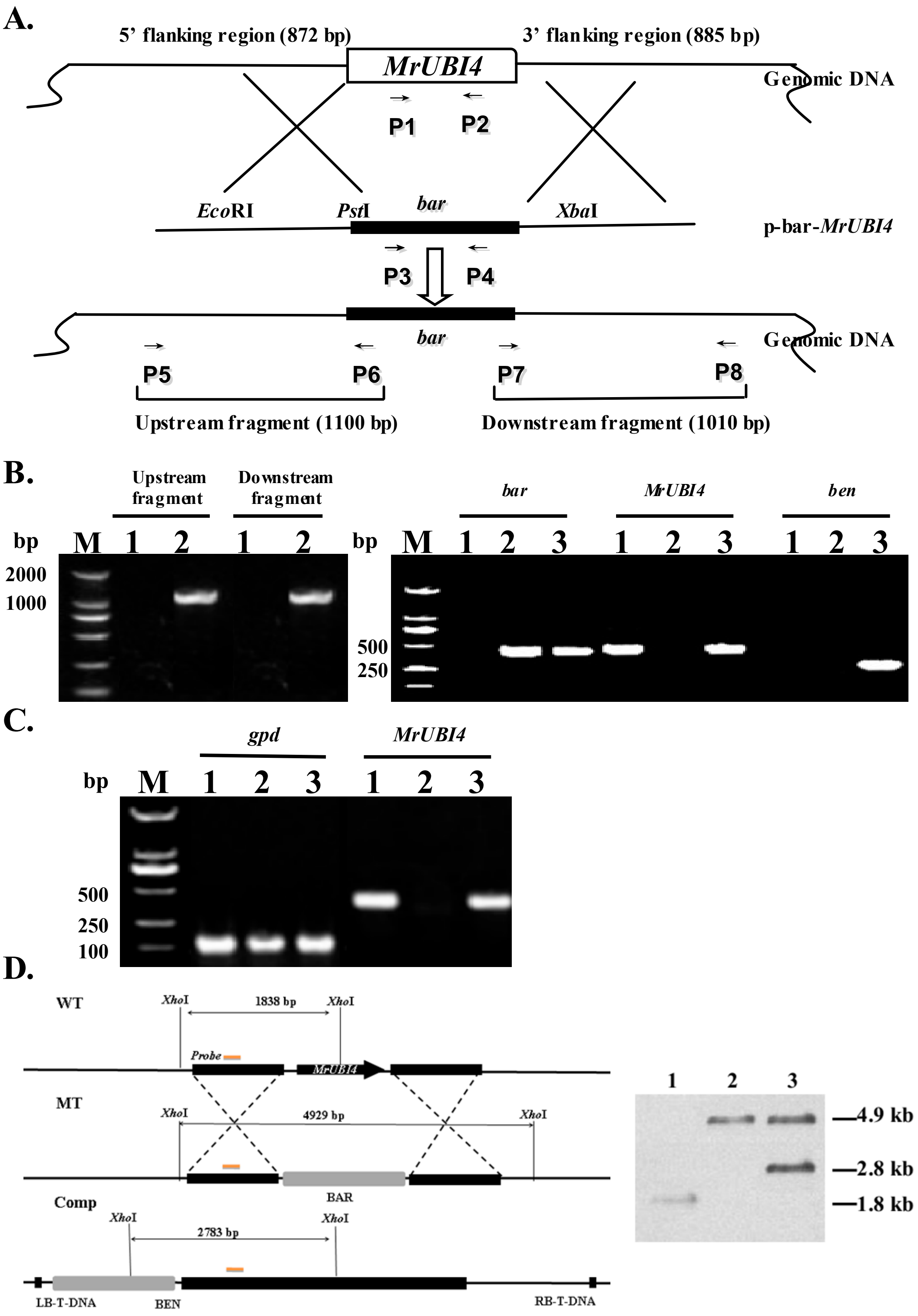

Figure S1. *MrUBI4* gene deletion in *M. robertsii*. (A) Schematic diagram of *MrUBI4* gene disruption based on the homologous recombination approach. The 1118-bp fragment (including the *MrUBI4* coding region) was replaced by the 942-bp *bar* cassette via homologous recombination of the overlapping regions of the 5' and 3' fragments in *MrUBI4* and p*bar-MrUBI4* (recombinant vector), respectively. The corresponding locations of the primers are shown with arrows. **(B)** PCR confirmation assay. Genomic DNAs extracted from the different strains were used as templates for

PCR. The upstream fragment, downstream fragment, *MrUBI4*, *bar*, and *ben* were amplified using the primer sets P5/P6, P7/P8, P1/P2, P3/P4, and *ben*-F/*ben*-R, respectively (Table 1). A P5/P6 and P7/P8 amplified 1100-bp and 1010-bp recombinant fragment is only present in the Δ*MrUBI4* mutant, and not in the WT (*left panel*). *MrUBI4* was amplified using the primer sets P1/P2 from WT and Comp, and not from Δ*MrUBI4*. The glufosinate-ammonium-resistance gene (*bar*) was amplified using the primer sets P3/P4 from the Δ*MrUBI4* and Comp mutants, and not from WT. The benomyl-resistance gene (*ben*) was amplified only with the primer sets *ben*-F/*ben*-R from Comp, and not from WT and Δ*MrUBI4* (*right panel*). M: Marker; 1: WT (wild-type strain), 2: MT (Δ*MrUBI4* strain), 3: Comp (complementary strain). **(C)** RT-PCR assay to verify *MrUBI4* for *MrUBI4* gene expression in the WT, Δ*MrUBI4*, and Comp strains. Using cDNA as the template, the *MrUBI4* partial sequence was amplified with the primer sets P1/P2 from WT and Comp, but not from Δ*MrUBI4*. Thus, our results show that the *MrUBI4* transcripts are completely undetectable in the Δ*MrUBI4* gene-deletion mutant; however, it is detectable in the WT and Comp strains. *gpd* was used as an internal control. M: Marker; 1: WT (wild-type strain), 2: MT (Δ*MrUBI4* strain), 3: Comp (complementary strain). **(D)** Southern blot assay. Schematic diagram of the enzymatic digestion of genomic DNAs from different strains (*left panel*) and hybridization (*right panel*). Southern blot assay was performed using *Xho*I-digested genomic DNAs from the WT, Δ*MrUBI4*, and Comp strains. As expected, WT showed a 1.8-kb fragment, whereas Δ*MrUBI4* showed a 4.9-kb fragment, which was also present in Comp (as it was generated based on Δ*MrUBI4*). An additional 2.8-kb fragment was also observed in Comp. The results of the Southern blot analysis indicate the integration of a single copy of the disruption cassette at the *MrUBI4* locus in the Δ*MrUBI4* strain. The blot was probed with the 5′ fragment of *MrUBI4*. Lane 1: WT (wild-type strain), Lane 2: MT (Δ*MrUBI4* strain), Lane 3: Comp (complementary strain).

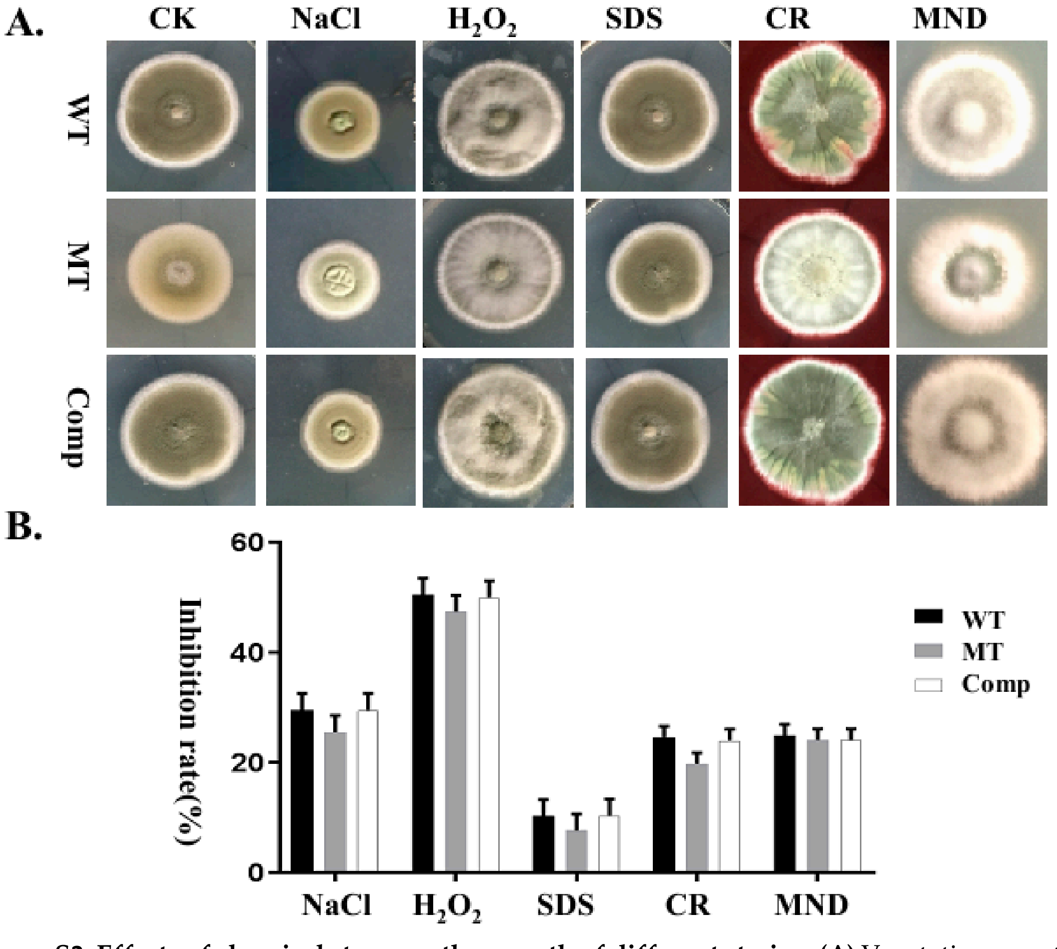

Figure S2. Effects of chemical stress on the growth of different strains. (A) Vegetative growth of the three strains on the PDA medium supplemented with different chemicals was compared after growing for 10 days at 25 °C. **(B)** Statistical analyses of the inhibition rates in the different chemical

stress assay. SDS: Sodium dodecyl sulfate, CR: Congo red, MND: menadione. WT: wild-type strain, MT: Δ*MrUBI4* strain, Comp: complemented strain.

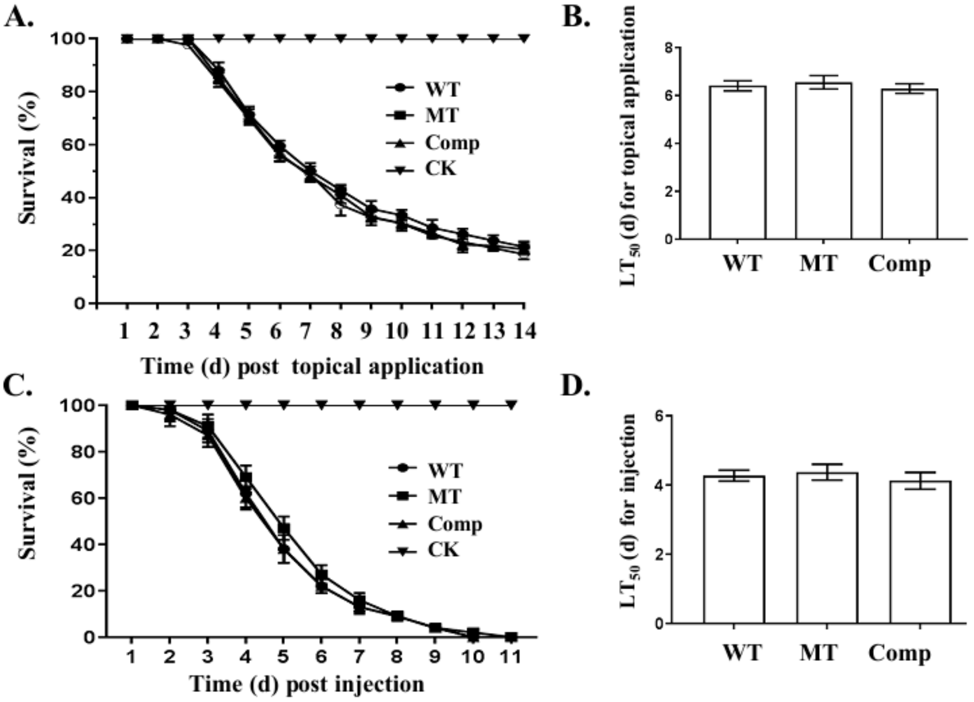

Figure S3. Fungal virulence assays of different *M. robertsii* strains. (A) Survival of *Galleria mellonella* after topical application of conidial suspension from WT, MT and Comp strains, respectively. Control insects were treated with sterile 0.05% Tween-80 solution. **(B)** LT_{50} (days) of the WT, MT and Comp strains after topical inoculation. **(C)** Survival of *G. mellonella* after injection of conidial suspension from WT, MT and Comp strains, respectively. Control insects were injected with 5 μl sterile H_2O. **(D)** LT_{50} (days) of the WT, MT and Comp strains after injection. WT: wild-type strain, MT: Δ*MrUBI4* strain, Comp: complemented strain, CK: control.

Table S1. Primers used for qRT-PCR analysis of conidiation-associated and heat stress-related genes

Gene	Accession number	Annotation	Sequence of primer sets (5′- 3′)
**Involved in conidiation**			
flbA	MAA_06313	Developmental regulator flbA	ACTCCAAAGGGCATCACG /CAACAAAGCGGCGGAATA
flbB	MAA_00196	BZIP-type transcription factor	TCCACGCTGCTTGATT /CCTCACTTTGCGACCC
flbC	MAA_08443	Putative zinc finger protein C	GCAGCATCACCACTACCT /TTAAACGGCTTCTCCC
flbD	MAA_03655	Conidiophore development protein	AACGATGGGCTGAGATTG /GGTGATTGAGTTTCGGATG
fluG	MAA_00122	protein fluG	TGCGGGTTGAATACGG /CTCCACCTCTTTCTCCTTGA
brlA	MAA_10599	C_2H_2 conidiation transcription factor BrlA	CAACAGCAGGAATCGC /GCTTATCGGCTGACTTTG
abaA	MAA_00694	Conidiation transcription factor AbaA	AAACCACTATTCCTGCTCC /AGCCTGCCTGTTACGATA
wetA	MAA_02845	Conidial maturation factor WetA	CGACGAAATAGGAAAGCA /TGAAGTGGAGGAGATACGG
vosA	MAA_05862	Protein VosA	ACCGAAATCGTGAGTG /GTTGCCCTTCTTGATG
StuA	MAA_02988	APSES transcription factor	GCAAGGCACCAACCCACT /TGCTGCTCCGTAGGCTGA
cag8	DQ826044.1	Conidiation-associated gene 8	AAGCTGATGGCTAGCGTAAG /TTGCGGTTGGAACGACTTTG
mero-Fus3	MAA_04503	Mitogen-activated protein kinase Mero-Fus3	GGCTCTAAAGCATCC /TCTTTGCTCAGGGTA
Pks1	MAA_07745	Polyketide synthases 1	AGGCGGCAATCCAGT /TGACGAAATCAAACAGC
Mlac1	MAA_07747	Laccase 1	GGGCCTCCTCGTATTTTGTC /GAAAGCGTCCTCAACCAGAC
**Involved in response to heat stress**			
hsp30a	MAA_07190	Heat shock protein 30	CTTCGCACAAGGCCACC / GAGAACTCGCCGACGCT
hsp30b	MAA_04014	Heat shock protein 30	CTGAGCCCGAGGAGAAG / GACACGGTTGGGAAAGT
hsp40a	MAA_02497	Heat shock protein 40	AGCGAGGCGAGGTGAA / GGTGGTGGCAGCATTT
hsp40b	MAA_03231	Heat shock protein 40	AGAGGAAGCCAACGACG / TCCGCAAGAGTAACAACGAG
hsp40c	MAA_06393	Heat shock protein 40	CGCCAACAGGAAAGACT / GCCGTGGTAAGAGGAAT
hsp60	MAA_07685	Heat shock protein 60	CGGCCAACTTTGACCAG / CAATGACAACGGAACCCT
hsp70b	MAA_00810	Heat shock protein 70	TTCAAAGAGGGATACCACC / CAACTTGTCGGAAGAATGA
hsp90	MAA_04726	Heat shock protein 90	TATGTCCGCCGTGTCTT / TGTTCTGCTGGAGGGTC
hsp104	MAA_03534	Heat shock protein 104	TCTGCGGTCCCTCTGGT / CGGCTAAGTGCGTGTCG
tps	MAA_04676	Trehalose-6-phosphate synthase	GTATGCCTCGTCTCCTCG / GCCAGTTCCTCCGTGTT
nth	MAA_04403	Neutral trehalase	CGAGCGTCTGGCCTACA / CCTTCTTTGGCAACACC

mtd	MAA_09034	Mannitol dehydrogenase	GTCATCACGGCGTCCAT / GAGGCCAGTGTCGATGTAG
mpd	MAA_08216	Mannitol-1-phosphate dehydrogenase	ACGAGTTTGAGGAGGATG / CGAGAAAGTTTACGCAGAG
tpp	MAA_09905	Trehalose-6-phosphate phosphorylase	CGTGCGGTCTTATTGTCC / CGTTTGAGGGATTGGATG
**Involved in response to DNA damage**			
wc	MAA_04453	white collar 1	CCTTATCCTCGGTTTG / CTTTGCTTCCGTCTTA
phr	MAA_01734	putative regulator of deoxyribodipyrimidine photo-lyase	CTCGTCGGTGTATCAA / TGGGCTCGGAGGTTTC
uve-1	MAA_09725	UV-endonuclease	GTCGCGTCCGCTGTTA / GCTTGCTCCTTGTCCT
